# Markers of Novelty Processing in Older Adults Are Stable and Reliable

**DOI:** 10.3389/fnagi.2019.00165

**Published:** 2019-06-28

**Authors:** Hura Behforuzi, Nicole C. Feng, Adam R. Billig, Eliza Ryan, Erich S. Tusch, Phillip J. Holcomb, Abdul H. Mohammed, Kirk R. Daffner

**Affiliations:** ^1^Laboratory of Healthy Cognitive Aging, Department of Neurology, Center for Brain/Mind Medicine, Brigham and Women’s Hospital, Harvard Medical School, Boston, MA, United States; ^2^Department of Psychology, San Diego State University, San Diego, CA, United States; ^3^Department of Psychology, Linnaeus University, Växjö, Sweden; ^4^Department of Neurobiology, Care Sciences and Society, Division of Neurogeriatrics, Karolinska Institutet, Stockholm, Sweden

**Keywords:** ERP, test-retest reliability, aging, novelty processing, visual modality

## Abstract

Exploratory behavior and responsiveness to novelty play an important role in maintaining cognitive function in older adults. Inferences about age- or disease-related differences in neural and behavioral responses to novelty are most often based on results from single experimental testing sessions. There has been very limited research on whether such findings represent stable characteristics of populations studied, which is essential if investigators are to determine the result of interventions aimed at promoting exploratory behaviors or draw appropriate conclusions about differences in the processing of novelty across diverse clinical groups. The goal of the current study was to investigate the short-term test-retest reliability of event-related potential (ERP) and behavioral responses to novel stimuli in cognitively normal older adults. ERPs and viewing durations were recorded in 70 healthy older adults participating in a subject-controlled visual novelty oddball task during two sessions occurring 7 weeks apart. Mean midline P3 amplitude and latency, mean midline amplitude during successive 50 ms intervals, temporospatial factors derived from principal component analysis (PCA), and viewing duration in response to novel stimuli were measured during each session. Analysis of variance (ANOVA) revealed no reliable differences in the value of any measurements between Time 1 and 2. Intraclass correlation coefficients (ICCs) between Time 1 and 2 were excellent for mean P3 amplitude (ICC = 0.86), the two temporospatial factors consistent with the P3 components (ICC of 0.88 and 0.76) and viewing duration of novel stimuli (ICC = 0.81). Reliability was only fair for P3 peak latency (ICC = 0.56). Successive 50 ms mean amplitude measures from 100 to 1,000 ms yielded fair to excellent reliabilities, and all but one of the 12 temporospatial factors identified demonstrated ICCs in the good to excellent range. We conclude that older adults demonstrate substantial stability in ERP and behavioral responses to novel visual stimuli over a 7-week period. These results suggest that older adults may have a characteristic way of processing novelty that appears resistant to transient changes in their environment or internal states, which can be indexed during a single testing session. The establishment of reliable measures of novelty processing will allow investigators to determine whether proposed interventions have an impact on this important aspect of behavior.

## Introduction

Participating in cognitively stimulating activities has been associated with a reduced risk of cognitive decline and dementia (Wilson et al., 2002; Gates et al., 2011; Najar et al., 2019). There has been a growing number of intervention studies aimed at engaging individuals in cognitively demanding activities. Curiosity/exploratory behavior and novelty seeking have been shown to be one of the driving forces that play an important role in maintaining cognitive function, learning, and even longevity in aging populations (Swan and Carmelli, 1996; Galli et al., 2018; Sakaki et al., 2018). Prior work in our laboratory has demonstrated that increased responsiveness to novelty is associated with successful cognitive aging (Daffner et al., 2006b; Riis et al., 2008). It is critical to establish reliable measures of novelty processing that will allow investigators to determine whether proposed interventions have an impact on this important aspect of behavior.

The process of orienting to and actively exploring novel events facilitates new learning and is an integral part of adapting to a rapidly changing environment (Sokolov, 1963; Daffner et al., 1998; Mesulam, 1998). The neural and behavioral underpinnings of novelty processing have been investigated using functional imaging [PET and functional magnetic resonance imaging (fMRI; Tulving et al., 1996; Opitz et al., 1999; Downar et al., 2000, 2002; Kiehl et al., 2001a,b; Bunzeck and Düzel, 2006; Bunzeck et al., 2007, 2010, 2012; Strobel et al., 2008; Blackford et al., 2010)], magnetoencephalography (Bunzeck et al., 2009; Naeije et al., 2016), and especially high temporal resolution event-related potentials (ERPs) that are often measured during different kinds of oddball paradigms (Näätänen, 1990; Fabiani and Friedman, 1995; Daffner et al., 1998, 2001, 2003; Friedman et al., 2001; Polich and Comerchero, 2003; Schomaker and Meeter, 2014; Kaufman et al., 2016b). Although the N1, P2, and N2 ERP components have been shown to be elicited by novel stimuli (Courchesne et al., 1975; Beck et al., 1980; Chong et al., 2008; Riis et al., 2008; Friedman et al., 2011; Tarbi et al., 2011; Barry et al., 2013; Schomaker et al., 2014), the novelty P3 component remains the most commonly employed ERP marker of novelty processing (Friedman et al., 2001).

The impact of normal aging and different neurological conditions on novelty processing has been an area of active investigation (Knight, 1984; Kaipio et al., 1999; Daffner et al., 2000a,b, 2001, 2003, 2006b; Stevens et al., 2007; Sokhadze et al., 2009; Ischebeck et al., 2011; Schott et al., 2015; Kaufman et al., 2016a; Sanjuan et al., 2018). Of note, inferences about age- or disease-related differences in neural and behavioral activity are most often based on results from single experimental testing sessions. There has been very limited research on whether such findings represent stable characteristics of the populations studied, which is essential if investigators are to draw appropriate conclusions about differences in response to novelty across diverse clinical groups or to determine the result of interventions aimed at promoting exploratory behaviors. The current study focuses on the stability and reliability of behavioral and ERP responses to novel stimuli in a sample of older adults who participated in a subject-controlled novelty oddball paradigm, as described below.

In the traditional version of the novelty oddball task, deviant stimuli are most commonly used to assess the degree to which participants are distracted from their assigned task, which is to identify (and often respond to) designated target stimuli (Fabiani and Friedman, 1995; Friedman et al., 2001; Polich and Comerchero, 2003; Kaufman et al., 2016b). Stimulus durations are fixed. By contrast, in the subject-controlled visual novelty oddball paradigm, participants determine viewing duration of stimuli by a button press (Daffner et al., 2006b; Chong et al., 2008). Viewing duration is used as an index of visual attention/exploratory behavior, and the P3 amplitude serves as an index of resources allocated to attentional processing (Berlyne, 1960; Daffner et al., 1994, 1998, 2000b). In this version of the paradigm, novel stimuli do not primarily serve as task-irrelevant distracters, but as potential “invitations” to explore interesting or salient aspects of one’s environment (Chong et al., 2008).

Based on investigations of patients with focal neurological lesions (Daffner et al., 2000a,b, 2003) who participated in a subject-controlled novelty oddball task, we have proposed that the prefrontal cortex and posterior parietal cortex reflect two nodes of a neuroanatomical network for responding to and processing of novelty (Daffner et al., 2003). The prefrontal cortex regulates the allocation of attentional resources to potentially significant events in the environment (Daffner et al., 2000a,b,d, 2003). The posterior parietal cortex is involved in updating one’s internal model of the environment to account for novel events (Daffner et al., 2003), a hypothesis consistent with Mesulam’s schema (Mesulam, 1981, 1990) of the posterior parietal cortex as a gateway to integrating information to develop a dynamic internal representation of the environment. Injury to this frontoparietal network is indexed by disruption of the novelty P3, which has been strongly linked to diminished attention to novel stimuli as measured by viewing duration (Daffner et al., 1998, 2000b,c, 2001). Also of note, we have shown that the P3 amplitude to novel visual stimuli in this paradigm inversely correlates with the degree of apathy in neurological patients, as measured by informant ratings (Daffner et al., 2000b, 2001). In addition, we have found that cognitively high performing older adults generate larger novelty P3 responses and spend more time attending to novel events than their cognitively average performing peers (Daffner et al., 2006b). Moreover, cognitively high performing older adults produce a larger P3 response to novel stimuli than their younger, matched cognitively high performing counterparts (Daffner et al., 2006a,b), which we have suggested represents successful compensatory activity adopted by these older adults.

In summary, the subject-controlled novelty oddball paradigm has provided an opportunity to examine the relationship between neural and behavioral responses to novel visual stimuli. Additionally, results in the lab have been associated with meaningful real-world behavior, specifically the degree of apathy displayed by neurological patients. Thus, it appears to be a promising paradigm to investigate the stability of the response to novelty in older adults.

ERP measures exhibit variability that can be due to a variety of sources (Segalowitz and Barnes, 1993) including biological and state factors such as arousal (Koshino et al., 1993), circadian rhythms and seasonal cycles (Deldin et al., 1994; Huang et al., 2006), exercise and fatigue (Yagi et al., 1999), sleep deprivation (Morris et al., 1992), and mood (Pierson et al., 1996; Cavanagh and Geisler, 2006). However, a fundamental tenant of research in this area is that ERP components are reliable markers of underlying cognitive operations and processes (Kappenman and Luck, 2012) that may differ across clinical populations. If so, ERP results should demonstrate relative consistency over time. Research in this area has tended to focus on test-retest reliability of the P3 response of young adults to target stimuli in the auditory modality (Sinha et al., 1992; Segalowitz and Barnes, 1993; Kinoshita et al., 1996; Sandman and Patterson, 2000; Walhovd and Fjell, 2002; Lew et al., 2007), with fewer studies examining this issue using paradigms in the visual modality (Sinha et al., 1992; Cassidy et al., 2012; Brunner et al., 2013; Huffmeijer et al., 2014). These studies have varied in terms of paradigms used and the intervals between test and retest. In general, the investigations have demonstrated that P3 latency and P3 amplitude values in normal individuals are relatively stable, with no significant differences between test and retest values at follow-up intervals that have varied between 2 days and 36 months. Test-retest reliability [as measured by Pearson’s r or intraclass correlation coefficient (ICC)] has ranged from 0.50 to 0.86 for P3 amplitude measures and from 0.40 to 0.88 for P3 latencies (Segalowitz and Barnes, 1993; Kinoshita et al., 1996; Sandman and Patterson, 2000; Walhovd and Fjell, 2002; Hall et al., 2006; Lew et al., 2007; Cassidy et al., 2012). Investigations of test-retest reliability of ERPs in older adults are particularly sparse (Sandman and Patterson, 2000; Walhovd and Fjell, 2002). These studies have reported lower reliability of latency in older individuals than young adults and greater reliability of amplitude than latency measures across all ages. No investigations seem to have highlighted novelty processing.

## Materials and Methods

### Participants

Healthy older subjects were recruited through community announcements in the Boston metropolitan area. All subjects provided written informed consent approved by the Partners Human Research Committee. Brigham and Women’s Hospital, where the study took place, is part of the Partners Healthcare system. To be included in this study, participants were required to be age 65 or older, English speaking, have a Mini-Mental State Exam (MMSE) (Folstein et al., 1975) score ≥26, an estimated IQ on the American National Adult Reading Test (AMNART) (Ryan and Paolo, 1992) ≥90, and score within 2 SDs of age-appropriate means on the short form of the Boston Naming Test (Kaplan et al., 1983) and on the Logical Memory Subtest of the Wechsler Memory Scale—Third Edition (Wechsler, 1997).

Subjects were excluded if they had a history of central nervous system (CNS) diseases or major psychiatric disorders based on the Diagnostic and Statistical Manual of Mental Disorders, 4th edition (DSM-IV) criteria (American Psychiatric Association, 1994), Geriatric Depression Scale (GDS) (Yesavage et al., 1982) score ≥10, corrected visual acuity worse than 20/50 (as tested using a Snellen eye chart), severe hearing impairment that would interfere with their ability to participate in the experiment or complete neuropsychological testing, a history of medical conditions that would limit their ability to participate in a physical exercise program, focal abnormalities on neurological examination consistent with a CNS lesion or a Clinical Dementia Rating Scale (Morris, 1993) score of 0.5 or above, based on interview questions and completion of a questionnaire by an informant who knew the subject well. See Table 1 for subject demographic information and neuropsychological test performance.

**Table 1:** Subject characteristics.

	Age (years)	Sex (Male/Female)	Education (years)	MMSE	AMNART	GDS
*N* = 70 Subjects	75.2 (6.5)	18/52	17.7 (2.9)	29.1 (1.2)	122.3 (5.6)	2.8 (2.2)

*Values are given as mean (standard deviation). MMSE, Mini Mental State Examination; AMNART, American Adult Reading Test; GDS, Geriatric Depression Scale*.

### Experimental Procedure

The experiment consisted of a subject-controlled visual novelty oddball task that has previously been used to study normal aging (Daffner et al., 2003; Riis et al., 2009) as well as patients with focal neurological injury due to a cerebral infarction (Daffner et al., 2000b, 2003) and patients with mild Alzheimer’s disease (Daffner et al., 2001). Alternate versions of the task were presented during two sessions approximately 7 weeks apart, the order of which varied randomly across subjects. Stimuli were presented using E-Prime software (E-Prime 2.0, 2012). There were three categories of visual stimuli: frequent standard stimuli (a triangle)-70% frequency, rare target stimuli (upside down triangle)-15% frequency, and rare novel stimuli (randomly drawn from a set of unfamiliar line drawings many of which came from the collection of drawings that have been used by Kosslyn et al., 1994 and Kroll and Potter, 1984)-15% frequency (each shown only once). Two-hundred and forty line drawings, white on black background, were presented in four blocks of 60, each at the center of a high-resolution computer monitor. Visual stimuli appeared one at a time within a fixation box, subtending a visual angle of ~3.5 × 3.5°, which remained on the screen at all times. Visual stimuli subtended an angle of ~2.75° along their longest dimension.

**Figure 1:**
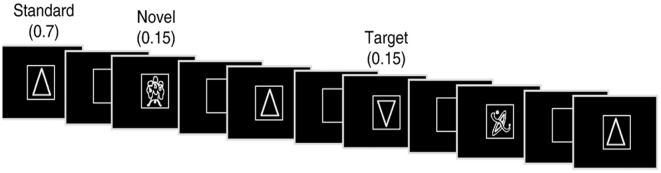
Illustration of an experimental run.

Subjects controlled viewing duration of stimuli by space bar press that triggered the onset of the next stimulus. Subjects also responded to designated targets with a mouse click. All stimuli were displayed for a minimum duration of 600 ms, regardless of when the subject pressed the space bar to ensure that each stimulus was visible when pertinent ERP components (e.g., P3) were elicited. The interstimulus interval ranged between 800 and 1,200 ms. Stimuli were presented in a pseudorandom order with the extra constraints that no more than two novel stimuli were shown successively, and that each block of 60 stimuli had the same number of standard stimuli and approximately the same number of target and novel stimuli. Each subject started the experiment after a series of practice runs that did not include novel stimuli. See Figure 1 for an illustration of the experiment.

Between sessions, subjects participated in one of four randomly assigned, structured programs involving adaptive or non-adaptive computerized cognitive training (CCT), physical exercise, or mindfulness meditation. Prior research suggests that CCT, physical exercise and mindfulness meditation may have a beneficial impact on cognitive functioning in older adults (Gates et al., 2011; Gard et al., 2014; Cheng, 2016; Tusch et al., 2016; Simon et al., 2018). Note that the purpose of these kinds of interventions has been to influence cognition, not novelty processing. Each intervention was structurally similar and conducted in subjects’ homes using interactive, web-based software over the course of 5 weeks (five sessions per week, ~35 min/session). The timing was based on the computerized Cogmed^®^ (Pearson Education, Inc., Fort Worth, TX, USA) program that offered an adaptive and non-adaptive training format. In the adaptive cognitive training program, task difficulty increased as training proceeded over time. In the non-adaptive cognitive training program, individuals participated in the same computerized program but with the same low-level task difficulty throughout the training period. In the mindfulness program, subjects participated in a series of mindfulness training and exercises where the tasks became increasingly more self-directed over the 5-week period. In the physical exercise training program participants were involved in a structured physical exercise program that aimed to progressively increase their level of activity over the training period. There was an approximately 1 week delay between the first ERP session and the start of each intervention and between the end of the intervention and the second ERP session. Thus, the duration between the experimental testing that took place at Time-1 and Time-2 was about 7 weeks (*M* = 7.2, SD = 1.2).

### ERP Recordings

An ActiveTwo electrode cap (Behavioral Brain Sciences Center, Birmingham, UK) was used to hold to the scalp a full array of 128 Ag-AgCl BioSemi (Amsterdam, Netherlands) “active” electrodes, whose locations were based on a pre-configured montage. Electrodes were arranged in equidistant concentric circles from 10 to 20 system electrode site Cz. In addition to the 128 electrodes on the scalp, six mini bio-potential electrodes were placed over the left and right mastoids (and used as references), beneath each eye and next to the outer canthi of the eyes to check for eye blinks and vertical and horizontal eye movements. EEG activity was digitized at a sampling rate of 512 Hz and filtered offline with a bandwidth of 0.016–100 Hz.

### Data Analysis

The focus of this report is on ERP and behavioral responses to novel visual stimuli.

#### Behavioral Data

E-Prime software was used to collect the behavioral data. Viewing durations were calculated by subtracting the stimulus onset time from the space bar press time. This measure served as an index of visual attention and exploratory behavior (Daffner et al., 1992, 2000b).

#### Average Waveforms

EEG data were analyzed using ERPLAB (Lopez-Calderon and Luck, 2014) and EEGLAB (Delorme and Makeig, 2004) toolboxes that operate within the MATLAB framework. Raw EEG data were resampled to 256 Hz and referenced off-line to the algebraic average of the right and left mastoids. EEG signals were filtered using an IIR bandpass filter with a bandwidth of 0.03–40 Hz (12 dB/octave roll-off). Eye artifacts were removed through an independent component analysis. Individual channels that upon visual inspection revealed a consistently different pattern of activity from surrounding channels were corrected with the EEGLAB interpolation function. EEG epochs for novel stimuli were averaged separately at three midline sites Fz, Cz, and Pz. The sampling epoch for each trial lasted for 1,200 ms, including a 200 ms pre-stimulus period that was used to baseline correct the ERP epochs. Trials were discarded from the analyses if they contained baseline drift or movement artifacts greater than 90 μV. Only trials with correct responses were included in the analyses. One of the 71 participants was excluded from further analyses because of excessively noisy ERP data, leaving a total of 70 participants.

P3 latency was measured as the local positive peak between 400 and 600 ms at midline electrodes Fz, Cz, and Pz in response to novel and target stimuli. P3 amplitude was measured as the average voltage between 400 and 600 ms at midline electrodes Fz, Cz, and Pz. Although the emphasis of this article is on the P3 response, a time course analysis to novel stimuli also was carried out by measuring the mean amplitude at Fz, Cz, and Pz for twenty 50 ms intervals across the entire 1,000 ms information processing period.

Statistical analysis of averaged ERP and behavioral data was carried out using IBM SPSS 25.0. In general, ERP dependent measures for novel and target stimuli were analyzed using repeated measures analysis of variance (ANOVA), with time (Time-1 vs. Time-2) and electrode site (Fz, Cz, and Pz) as the within-subjects variables and intervention condition (non-adaptive cognitive training, adaptive cognitive training, physical exercise, mindfulness training) as the between-subjects variable. The Greenhouse-Geisser correction was applied to all repeated measures with greater than 1 degree of freedom.

#### Principal Component Analysis

In addition to measuring average waveforms at midline electrodes, we performed a principal component analysis (PCA) of the data collected at Time-1 and Time-2 to identify and disentangle the constituent temporal and/or spatial components for further analysis of stability of ERP data over time. We used temporospatial PCA, following a method developed by Dien (2012). PCA is a data-driven method that decomposes ERP waveforms into their underlying components and is particularly useful in separating spatially and/or temporally overlapping components. Temporospatial PCA takes advantage of this method’s ability to parse components both temporally and spatially by breaking down each temporal principal component into a series of spatially distinct components.

Following the recommendation of Dien (2012), a temporospatial PCA was conducted on averaged trials for each individual subject at all 134 electrode sites at Time-1 and Time-2. ERPs to novel, target, and standard stimuli were included in the analysis to augment variance. Each dataset consisted of 307 time points between −200 and 1,000 ms. Utilizing the ERP PCA toolkit 2.38 (Dien, 2010), temporal PCA followed by a spatial PCA (on each identified temporal factor) was performed. A parallel test was used to restrict the number of factors generated for each PCA. The covariance matrix was used as input, with Kaiser normalization, followed by Promax rotation.

#### Intraclass Correlation

ICC represents the consistency of a measure with the time of testing introduced into the error variance (Shrout and Fleiss, 1979). ICC was used in the analysis of viewing duration, averaged ERP waves, and PCA components at the two time points. Per the descriptions and guidelines of different models of ICC (Shrout and Fleiss, 1979; Koo and Li, 2016), test-retest reliability was calculated by ICC method using a two-way mixed effect model with the setting of absolute agreement in SPSS. Since the values studied represented the average of multiple trials, average rather than single value ICC measurements are reported. As per classification of Cicchetti (Cicchetti, 1994), values less than 0.4 are indicative of poor reliability, values between 0.4 and 0.59 indicate fair reliability, values between 0.6 and 0.74 denote good reliability, and values greater than 0.75 are considered excellent reliability.

## Results

### Viewing Duration

**Figure 2:**
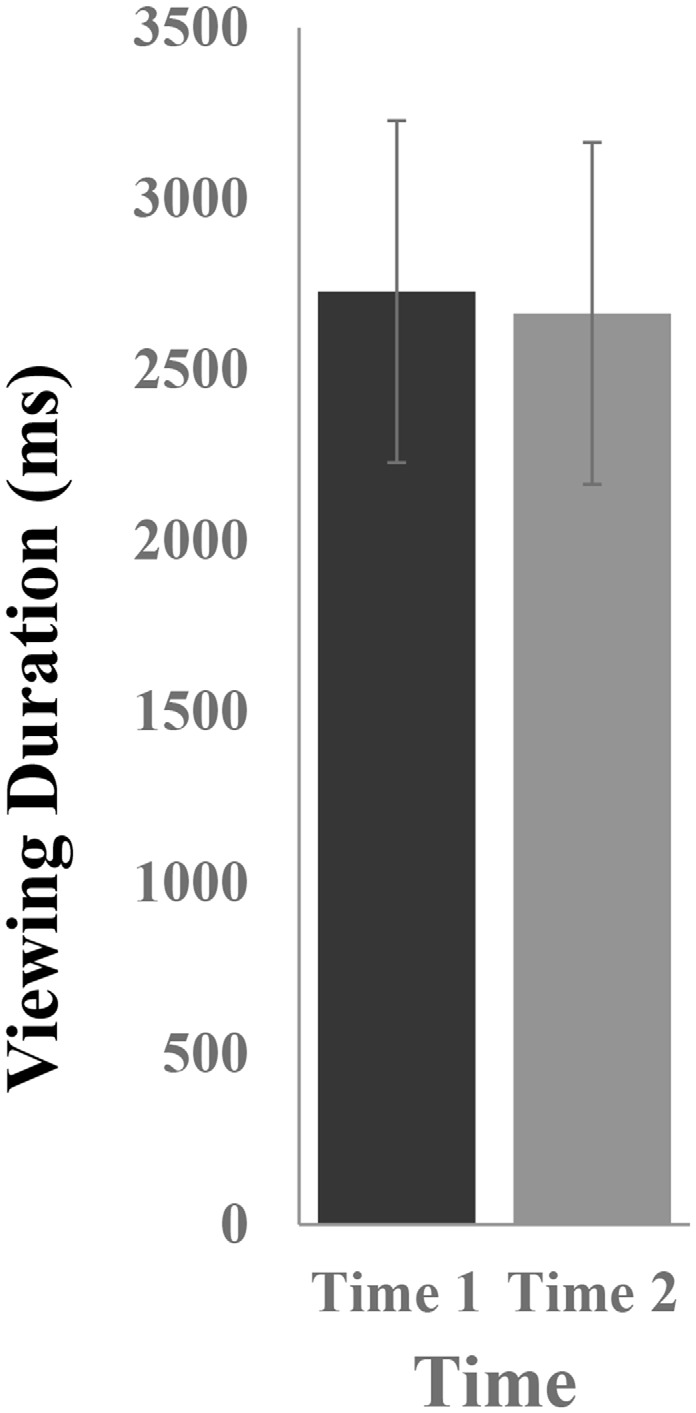
Mean [± standard error of the mean (SEM)] viewing duration (in ms) in response to novel stimuli.

Figure 2 illustrates the mean viewing durations in response to novel stimuli at Time-1 vs. Time-2. Repeated measures ANOVA was performed for the effect of time (Time-1, Time-2) on viewing duration. It demonstrated no effect of time, *F*_(1,66)_ = 0.06, *p* = 0.80, partial *η*^2^ = 0.0001. The average measure ICC between viewing duration at Time-1 and Time-2 was 0.81, with a 95% confidence interval from 0.69 to 0.88, *F*_(69,69)_ = 5.13, *p* < 0.001.

### Grand Average Waveforms

#### Novel Stimuli

**Figure 3:**
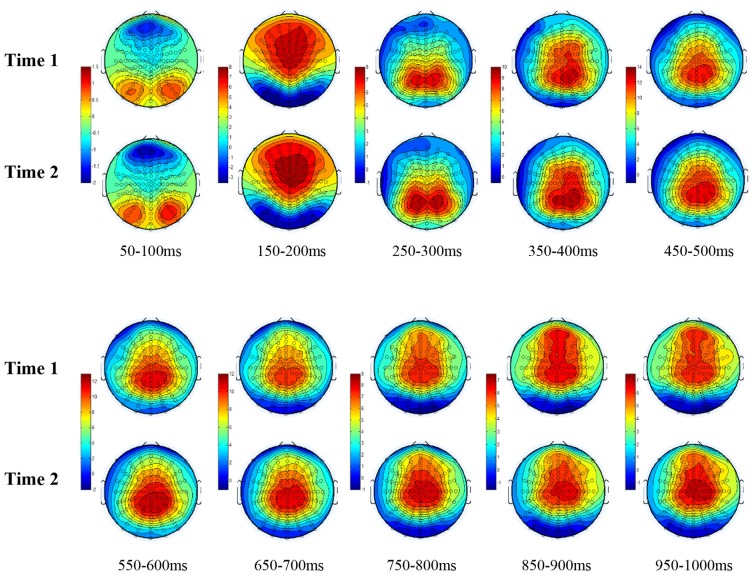
Topographic maps of the mean amplitude for every other 50 ms interval (beginning 50–100 ms) of novelty processing at Time-1 vs. Time-2 (Note that the scales are different across the time frames).

All the results are collapsed across the four structured programs since none of the findings were modulated by this between-subject variable. Figure 3 presents topographic surface potential maps in response to novel stimuli for Time-1 vs. Time-2 at 50 ms intervals. Note that the pattern of electrophysiologic response is very similar across the two time points.

**Figure 4:**
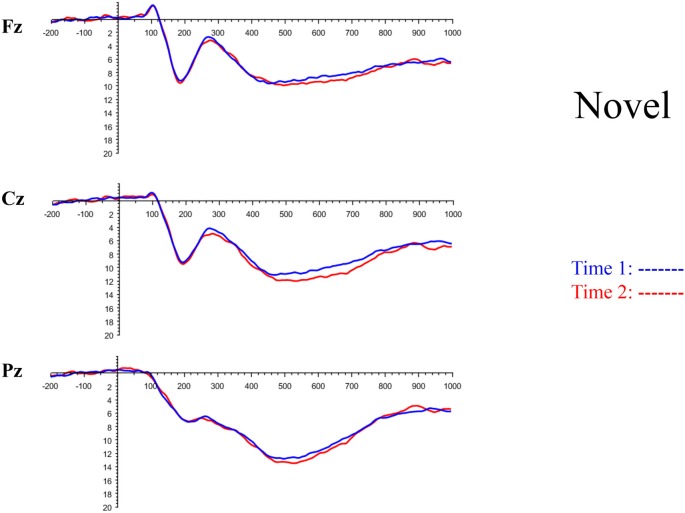
Grand average event-related potential (ERP) plots in response to novel stimuli at Fz, Cz, and Pz at Time-1 and Time-2.

**Figure 5:**
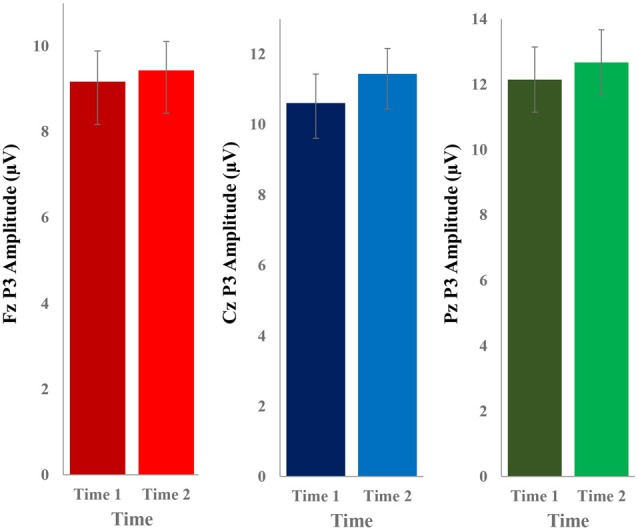
Bar graph of the mean (± SEM) P3 amplitude (in μV) in response to novel stimuli at Fz, Cz, and Pz at Time-1 and Time-2.

The grand average ERP plots for novel stimuli (Time-1 and Time-2) at midline electrode sites Fz, Cz, and Pz are presented in Figure 4. Figure 5 illustrates a bar graph of the mean P3 amplitude data at midline sites for novel stimuli. Repeated measures ANOVA for the P3 mean amplitude demonstrated no effect of time, *F*_(1,69)_ = 1.19, *p* = 0.28, partial *η*^2^ = 0.02; and no time × electrode site interaction, *F*_(2,138)_ = 1.68, *p* = 0.19, partial *η*^2^ = 0.02. There was an effect of electrode site on P3 mean amplitude, *F*_(2,138)_ = 41.6, *p* < 0.001, partial *η*^2^ = 0.38. *Post hoc* comparisons using the LSD test indicated that the P3 mean amplitude at Fz (*M* = 9.30 μV, SE = 0.65) was smaller than at Cz (*M* = 11.02 μV, SE = 0.73), which in turn was smaller than at Pz (*M* = 12.40 μV, SE = 0.71). The ICC between P3 mean amplitude collapsed across midline sites at Time-1 and Time-2 was 0.86, with a 95% confidence interval from 0.78 to 0.92, *F*_(69,69)_ = 7.31, *p* < 0.001.

Repeated measures ANOVA for P3 peak latency demonstrated no effect of time, *F*_(1,69)_ = 1.11, *p* = 0.30, partial *η*^2^ = 0.02; and no time × electrode site interaction, *F*_(2,138)_ = 0.18, *p* = 0.80, partial *η*^2^ = 0.003. There was an effect of electrode site on P3 peak latency, *F*_(2,138)_ = 5.00, *p* = 0.01, partial *η*^2^ = 0.07. *Post hoc* comparisons using the LSD test indicated that the mean P3 peak latency was longer at Cz (*M* = 510 ms, SE = 5.55) than Fz (*M* = 497 ms, SE = 5.24). There was no difference between Pz (*M* = 504 ms, SE = 5.52) and the other two electrode sites. The ICC between the average midline P3 peak latency at Time-1 and Time-2 was 0.56, with a 95% confidence interval from 0.30 to 0.73, *F*_(69,69)_ = 2.29, *p* < 0.001.

**Figure 6:**
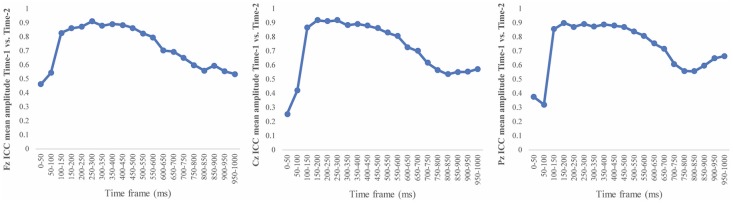
Intraclass correlation coefficient (ICC) between Time-1 and Time-2 of the mean amplitude for each 50 ms post novel stimuli at Fz, Cz, and Pz.

Figure 6 depicts the ICCs between the average amplitudes at Time-1 vs. Time-2 for each 50 ms interval in response to novel stimuli at Fz, Cz, and Pz. Except for two time frames (0–50 ms at Cz and 50–100 ms at Pz) the ICCs were significant throughout the 1,000 ms temporal epoch at midline electrodes. The ICC reliability ranged from 0.53 to 0.91 between 100 ms and 1,000 ms, with the very high reliabilities between 200 ms and 600 ms (ICC range 0.80–0.91).

#### Target Stimuli

**Figure 7:**
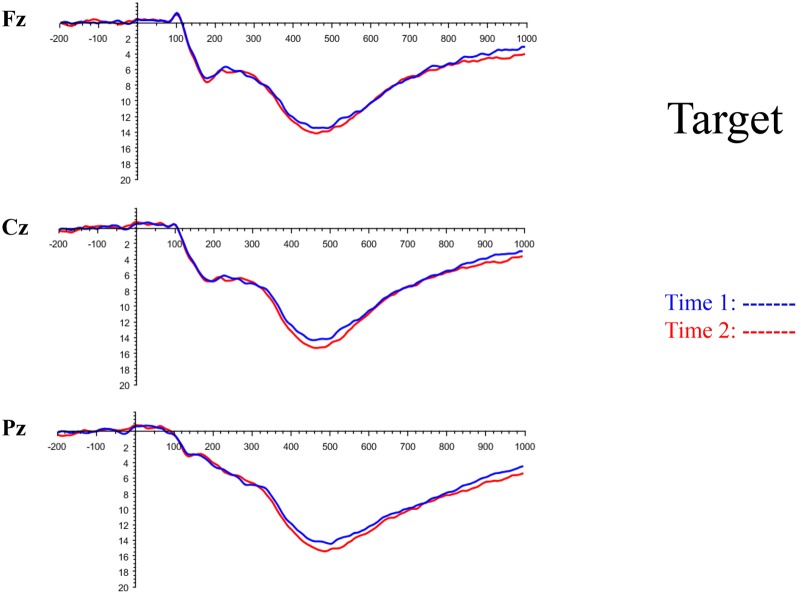
Grand average ERP plots in response to target stimuli at Fz, Cz, and Pz at Time-1 and Time-2.

Although the focus of this study was on novelty processing, data for target stimuli were also analyzed to help determine the consistency of response to non-novel visual stimuli. The grand average ERP plots for target stimuli (Time-1 and Time-2) at midline electrode sites Fz, Cz, and Pz are presented in Figure 7. Repeated measures ANOVA for target P3 amplitude demonstrated no effect of time, *F*_(1,69)_ = 3.29, *p* = 0.07, partial *η*^2^ = 0.05; and no time × electrode site interaction, *F*_(2,138)_ = 0.57, *p* = 0.55, partial *η*^2^ = 0.008. There was an effect of electrode site, *F*_(2,138)_ = 4.40, *p* = 0.02, partial *η*^2^ = 0.06. *Post hoc* comparisons using the LSD test indicated that the mean P3 mean amplitude at Fz (*M* = 12.65 μV, SE = 0.59) was smaller than at Cz (*M* = 13.43 μV, SE = 0.74) and Pz (*M* = 13.88 μV, SE = 0.72), with no difference between the latter two electrode sites. The ICC between P3 mean amplitude collapsed across midline sites at Time-1 and Time-2 was 0.90, with a 95% confidence interval from 0.84 to 0.94, *F*_(69,69)_ = 10.63, *p* < 0.001.

For P3 latency, repeated measures ANOVA revealed no effect of time, *F*_(1,69)_ = 1.22, *p* = 0.27, partial *η*^2^ = 0.01; and no time × electrode site interaction, *F*_(2,138)_ = 0.21, *p* = 0.76, partial *η*^2^ = 0.003. There was an effect of electrode site on P3 peak latency, *F*_(2,138)_ = 7.54, *p* = 0.002, partial *η*^2^ = 0.09. *Post hoc* comparisons using the LSD test indicated that the mean P3 peak latency at Pz (*M* = 493 ms, SE = 5.30) was longer than at Fz (*M* = 483 ms, SE = 5.61) or Cz (*M* = 480 ms, SE = 5.27), with no difference between the latter two electrode sites. The ICC between P3 peak latency collapsed across midline sites at Time-1 and Time-2 was 0.78, with a 95% confidence interval from 0.65 to 0.86, *F*_(69,69)_ = 4.59, *p* < 0.001.

### Novel vs. Target

To determine whether responses to novels differed from those to targets, the stimuli were compared to each other. Differences in viewing duration of novel vs. target stimuli were examined. Repeated measures ANOVA was performed with stimulus type (novel, target) and time (Time-1, Time-2) as within-subject variables. There was an effect of stimulus type, *F*_(1,66)_ = 8.11, *p* = 0.006, partial *η*^2^ = 0.11, due to longer mean viewing durations on novels (*M* = 2,725 ms, SE = 326) than targets (*M* = 1,852 ms, SE = 93.5). There was no effect of time, *F*_(1,3)_ = 0.63, *p* = 0.43, partial *η*^2^ = 0.01, and no time × stimulus type interaction, *F*_(1,66)_ = 0.08, *p* = 0.78, partial *η*^2^ = 0.001.

To assess P3 amplitude, repeated measures ANOVA was performed, with stimulus type (novel, target), time (Time-1, Time-2) and electrode site (Fz, Cz, and Pz) as within-subject variables. There was an effect of stimulus type, *F*_(1,69)_ = 23.4, *p* < 0.001, partial *η*^2^ = 0.25, due to the mean P3 mean amplitude to targets (*M* = 13.32 μV, SE = 0.64) being larger than to novels (*M* = 10.91 μV, SE = 0.67). There was no effect of time, *F*_(1,69)_ = 3.42, *p* = 0.07, partial *η*^2^ = 0.05, and no time × stimulus type interaction, *F*_(1,69)_ = 0.09, *p* = 0.77, partial *η*^2^ = 0.001. There was a stimulus type × electrode site interaction, *F*_(2,138)_ = 22.9, *p* < 0.001, partial *η*^2^ = 0.25. As noted above, the P3 mean amplitude to novel stimuli at Fz (*M* = 9.30 μV, SE = 0.65) was smaller than at Cz (*M* = 11.02 μV, SE = 0.72), which in turn was smaller than at Pz (*M* = 12.40 μV, SE = 0.71). In contrast, the P3 mean amplitude to target stimuli at Fz (*M* = 12.65 μV, SE = 0.59) was smaller than at Cz (*M* = 13.43 μV, SE = 0.73) and Pz (*M* = 13.88 μV, SE = 0.72), with no difference between the latter two electrode sites.

P3 latency was examined using repeated measures ANOVA, with stimulus type (novel, target), time (Time-1, Time-2) and electrode site (Fz, Cz, and Pz) as within-subject variables. There was an effect of stimulus type, *F*_(1,69)_ = 9.27, *p* = 0.003, partial *η*^2^ = 0.12, with P3 latency to novels (*M* = 504 ms, SE = 4.87) being longer than to targets (*M* = 486 ms, SE = 5.0). There was no effect of time, *F*_(1,69)_ = 0.04, *p* = 0.84, partial *η*^2^ = 0.001. There was also significant electrode × stimulus type interaction, *F*_(2,138)_ = 6.61, *p* = 0.003, partial *η*^2^ = 0.09. The mean P3 peak latency in response to novel stimuli was longer at Cz (*M* = 510 ms, SE = 5.55) than Fz (*M* = 497 ms, SE = 5.16). There was no difference between Pz (*M* = 504 ms, SE = 5.52) and the other two electrode sites. In contrast, the mean P3 peak latency in response to target at Pz (*M* = 493 ms, SE = 5.30) was longer than at Fz (*M* = 483 ms, SE = 5.61) or Cz (*M* = 480 ms, SE = 5.26), with no difference between the latter two electrode sites.

### PCA

A temporospatial PCA of the whole data set yielded 132 factor combinations [12 temporal factors (TFs), each with 11 spatial factors (SFs)]. Table 2 illustrates temporospatial factors that each accounted for >1% of the variance, ordered by the amount of variance explained by each factor. The table includes the factor name, peak latency, percentage of the total variance accounted for, topography at Time-1 and Time-2 in response to novel stimuli, and ICC (and *p*-values) between Time-1 and Time-2. One-hundred and twenty of the factor combinations were not analyzed further because each accounted for <1% of the total variance.

**Table 2:** Temporospatial factors accounting for >1% of variance.

Temporal spatial factor	Amount of variance explained	Peak latency	Waveform	Topography Time-1	Topography Time-2	Intraclass correlation *P*-value
TF02SF01	22.5%	389 ms	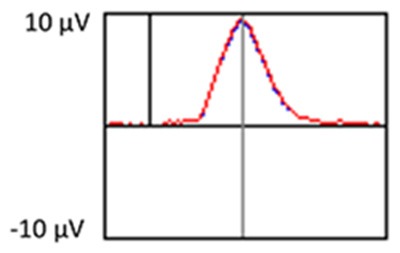	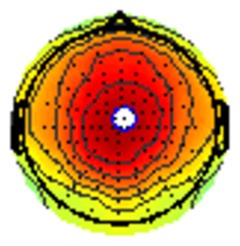	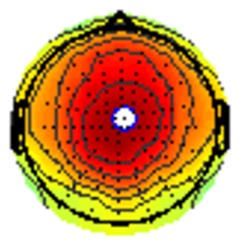	0.88 *P* < 0.001
TF01SF01	16.4%	819 ms	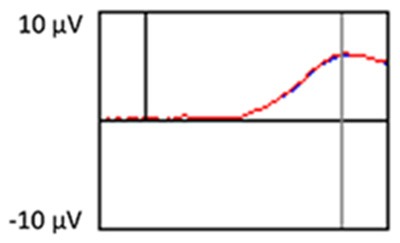	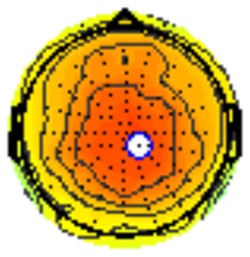	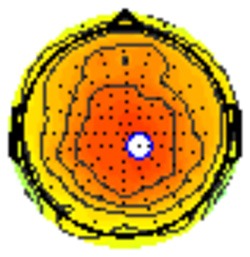	0.49 *P* < 0.01
TF03SF01	9.0%	573 ms	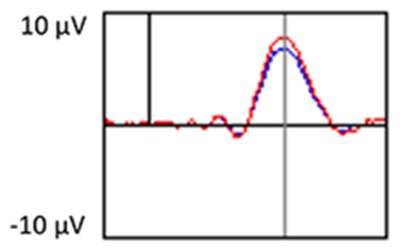	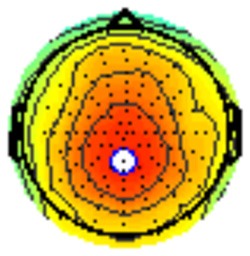	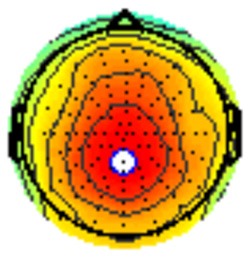	0.76 *P* < 0.001
TF02SF02	4.24%	389 ms	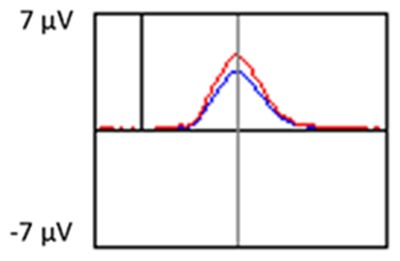	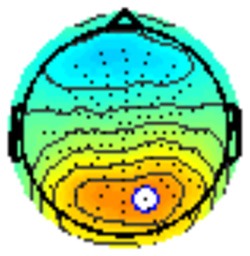	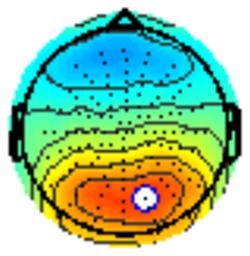	0.91 *P* < 0.001
TF01SF02	4.06%	819 ms	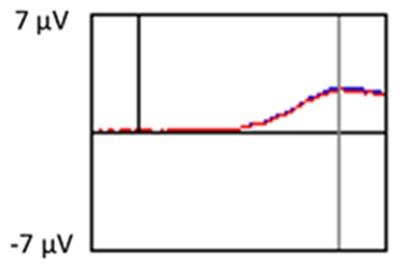	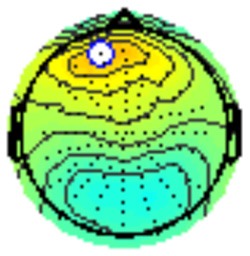	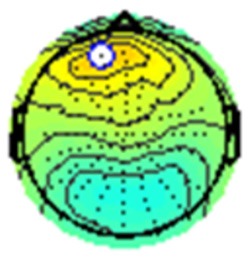	0.74 *P* < 0.001
TF04SF01	3.00%	167 ms	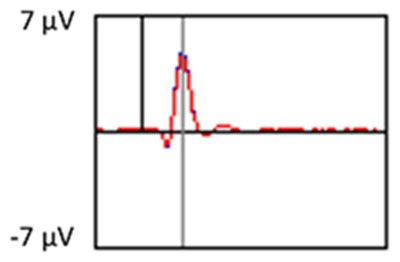	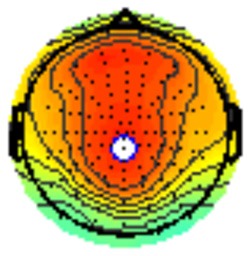	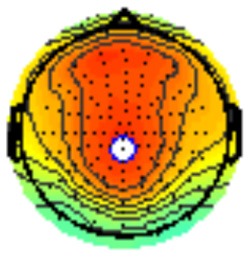	0.90 *P* < 0.001
TF05SF01	2.27%	221 ms	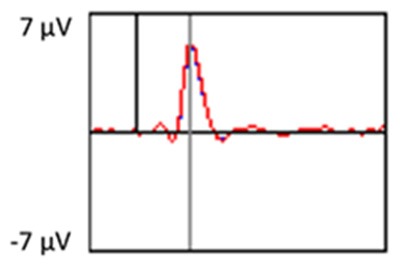	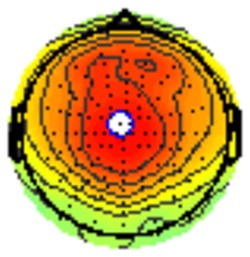	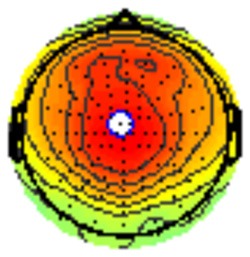	0.76 *P* < 0.001
TF01SF03	2.1%	819 ms	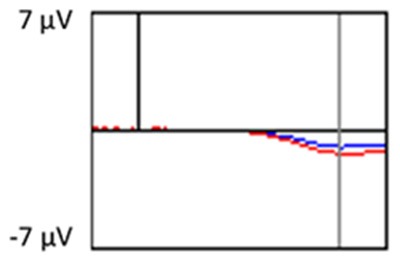	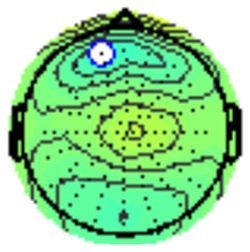	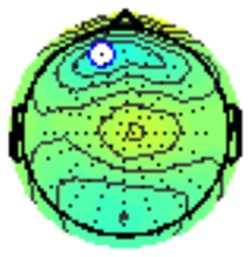	0.68 *P* < 0.001
TF03SF02	1.99%	573 ms	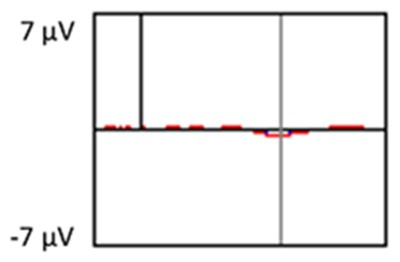	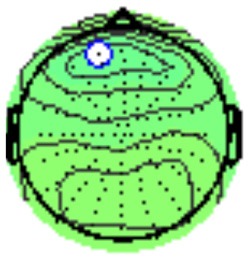	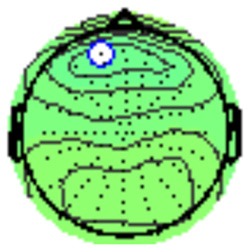	0.73 *P* < 0.001
TF06SF01	1.8%	303 ms	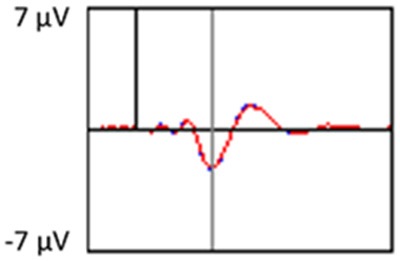	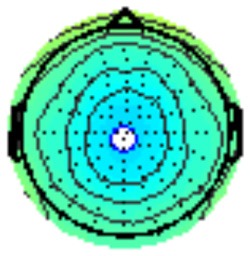	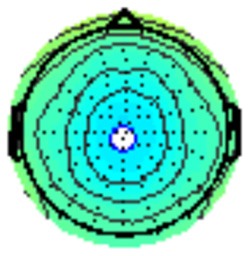	0.79 *P* < 0.001
TF01SF04	1.49%	819 ms	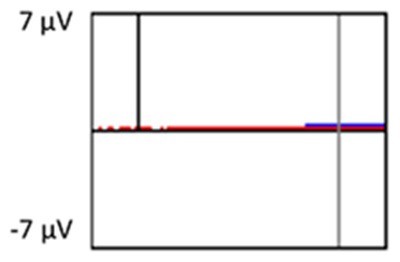	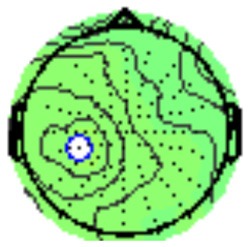	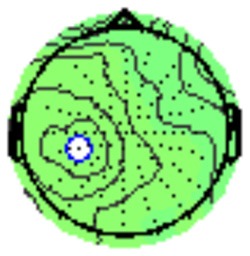	0.67 *P* < 0.001
TF01SF05	1.21%	819 ms	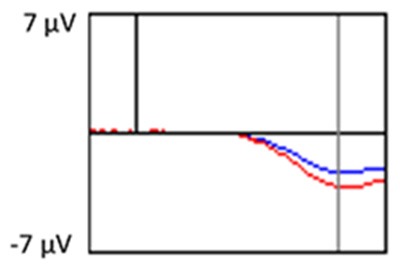	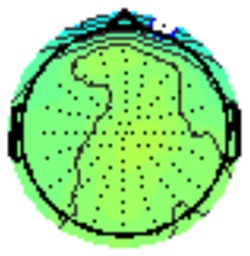	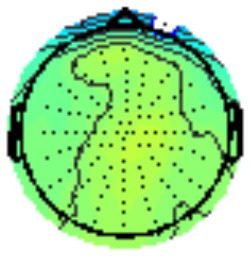	0.70 *P* < 0.001

(Note that the amplitude scales used differ across factors)

Based on visual inspection of the timing and topographic distribution of temporospatial factors, two factors, TF2SF1 (389 ms peak) and TF3SF1 (573 ms peak) likely contributed to the P3 component (Spencer et al., 1999, 2001; Goldstein et al., 2002; Dien et al., 2003). Both demonstrated excellent test-retest stability, with ICCs of 0.89 and 0.77 respectively. TF1SF1 (819 ms peak) was consistent with the late positive slow wave (Spencer et al., 2001; Alperin et al., 2015). TF4SF1 (167 ms peak) was suggestive of an early anterior P2, and TF5SF1 (221 ms peak) was consistent with late anterior P2 (Riis et al., 2009; Alperin et al., 2015). All but one temporospatial factor demonstrated good to excellent test-retest stability. TF1SF1 showed fair stability (see Table 2).

## Discussion

Attention to novel stimuli plays a critical role in adaptation, learning, and the maintenance of cognitive functions as adults grow older (Sokolov, 1963; Daffner et al., 1994, 2006b; Riis et al., 2008). ERPs have helped to track neurophysiological changes associated with novelty processing across different age groups and neurological conditions (Daffner et al., 2000a,b, 2003, 2006a,b). Using ERPs to characterize differences between clinical populations or to assess the impact of interventions on promoting engagement with one’s environment requires a demonstration of the reliability of the measures themselves. Much more research has been directed at investigating the consistency of ERP responses across testing sessions among young than old adults, and in response to target rather than novel events. The current study aimed to evaluate the test-retest reliability of electrophysiological and behavioral responses to novel stimuli in cognitively normal older adults.

Novel visual stimuli were infrequent and highly unusual/unfamiliar figures. Because participants in this subject-controlled novelty oddball paradigm had to determine the duration of each stimulus, the novel events were not task-irrelevant, as they are in traditional novelty oddball paradigms. In our study, there was electrophysiological and behavioral evidence that novel stimuli were processed differently from target ones. Viewing duration was much longer on novel than target events; P3 mean amplitude was larger in response to target than to novel stimuli; and P3 peak latency was longer to novel than target stimuli.

ANOVA yielded no reliable differences in the electrophysiologic and behavioral responses to novel visual stimuli between test sessions approximately 7 weeks apart, with *p*-values ranging from 0.27 (mean P3 amplitude) to 0.8 (viewing duration). These results point to the stability of the measures used. However, confirming the null hypothesis (i.e., no differences between sessions) is not possible statistically. Thus, we used ICC as a measure of test-retest reliability.

Our findings indicate that the mean P3 amplitude response to novel visual stimuli, as measured on average waveforms at midline sites, exhibits excellent reliability (ICC of 0.86). Converging evidence for the stability of the P3 to novel stimuli was derived from PCA, a data-driven method. The amplitude of the temporospatial factors consistent with the P3 components (TF2SF1 and TF3SF1) also demonstrated excellent reliability (ICC of 0.88 and 0.76, respectively).

In keeping with other reports in the literature on ERP latencies, P3 peak latency in response to novel visual stimuli demonstrated only fair reliability (ICC of 0.56) across the 7-week interval. P3 latency, a marker of processing speed, may be more sensitive than P3 amplitude to a variety of state functions, including level of arousal, variation in sleep, or changes in mood (Bruder et al., 1991; Polich and Kok, 1995; Polich, 2004). Latency measures are often reported to have lower test-retest reliability than amplitude measures regardless of age group (Sinha et al., 1992; Sandman and Patterson, 2000; Walhovd and Fjell, 2002; Olvet and Hajcak, 2009; Weinberg and Hajcak, 2011; but see Segalowitz and Barnes, 1993; Brunner et al., 2013 for conflicting evidence). Walhovd and Fjell (2002) found in a two-stimulus auditory oddball task that test-retest reliability of P3 latency was lower in older than younger adults. In contrast, these investigators and others (Hämmerer et al., 2013) who have used tasks in the visual modality have reported no differences in the reliability of P3 amplitude across age groups.

Our results also strongly point to stability in the electrophysiologic response to novel stimuli throughout the 1,000 ms temporal epoch studied and not only the interval containing the P3 component. Inspection of the surface potential maps for Time-1 vs. Time-2 (Figure 3) suggests considerable overlap in the appearance of scalp voltage distributions from 100 to 1,000 ms. This impression was validated by assessing the mean amplitude at midline electrode sites using time course analysis during sequential 50 ms intervals. This evaluation demonstrated fair to excellent reliability (ICC range 0.53–0.91) between 100 ms and 1,000 ms time range, with very high reliability between 200 ms and 600 ms (ICC range 0.80–0.91), which includes the temporal interval of the P3 component (see Figure 6). Moreover, with only one exception the 12 temporospatial factors analyzed (all of which peaked between 167 and 819 ms) demonstrated ICCs in the good to excellent range. Thus, we provide strong converging evidence in older adults for the stability of electrophysiological responses to novel stimuli throughout the measured information processing stream. These results of our study are consistent with findings reported by Walhovd and Fjell in their study using a two-stimulus auditory oddball task (Walhovd and Fjell, 2002). They investigated the reliability of successive 15 ms time window measurements across 0–705 ms post-stimulus and observed high reliability, especially during the temporal windows in which ERP components (N1, P2, P3) are conventionally measured. They suggest that these results may provide further validation of established ERP components as reflecting stable cerebral responses to different stimulus types.

In the current study, viewing duration was used as an index of visual attention and exploratory behavior (Daffner et al., 1994). Viewing duration of novel stimuli demonstrated excellent test-retest reliability (ICC of 0.81) over the 7-week period. This result suggests that an older individual may exhibit a characteristic degree of engagement by novel visual stimuli that remain stable over time. Both P3 amplitude and viewing duration can be understood in terms of resources being allocated in response to a presented stimulus (Daffner et al., 2000c). Both experimental measures appear to be consistent and reliable, a result that has notable implications for future research. The finding suggests that if a clinical intervention (behavioral or pharmacologic) is associated with a significant alteration in P3 amplitude or viewing duration in response to novel stimuli, it is unlikely that such changes would be simply due to chance. This idea is important because of interest in developing interventions to help older adults become more engaged by the novel aspects of their environment as a means of promoting healthy cognitive/brain aging (Wilson et al., 2002; Daffner et al., 2006b; Veyrac et al., 2009). Objective laboratory measurements of such engagement can serve as a valuable component of the research.

The generalizability of our findings remains uncertain. The participants in our study were well educated and had above average intellectual capacity. Further research is necessary to determine whether similar stability of electrophysiologic and behavioral responses to novelty would be observed in older adults with different demographic characteristics. It would be informative for future studies to include a sample of younger adults to help determine if there are age-related differences in test-retest reliability in response to novel visual events. Additional studies are also needed to address the reliability of ERP measures over periods longer than 7 weeks and across multiple testing sessions. The limited number of studies that have investigated the test-retest reliability over more than two sessions, with inter-session intervals ranging from days to months (Kinoshita et al., 1996) or even years (Sandman and Patterson, 2000) have provided additional support for the stability of ERP measurements.

## Conclusion

Older adults exhibit considerable stability in their electrophysiological and behavioral responses to novel visual events over a 7-week period. These results suggest older adults may have a characteristic way of processing novelty that appears resistant to transient changes in their environment or internal states, such as level of arousal, and that can be indexed during a single testing session.

## Data Availability

The datasets generated for this study are available on request to the corresponding author.

## Ethics Statement

This study was carried out in accordance with the recommendations of the Human Research Committee of the Partners Health Care system, with written informed consent from all subjects. All subjects gave written informed consent in accordance with the Declaration of Helsinki. The protocol was approved by the Partners Human Research Committee.

## Author Contributions

HB analyzed the data, wrote the initial manuscript, prepared the figures. NF analyzed the data, helped prepare the figures, and edited the manuscript. AB analyzed the data and edited the manuscript. ER worked with participants and helped collect the data. ET collected the data and assisted with analysis. PH helped design the experiment and interpret the ERP data. AM helped design the overall study. KD was responsible for the overall design of the experiment, the data analysis, and the final manuscript.

## Conflict of Interest Statement

The authors declare that the research was conducted in the absence of any commercial or financial relationships that could be construed as a potential conflict of interest.
